# Chloroplast NADPH-Dependent Thioredoxin Reductase from *Chlorella vulgaris* Alleviates Environmental Stresses in Yeast Together with 2-Cys Peroxiredoxin

**DOI:** 10.1371/journal.pone.0045988

**Published:** 2012-09-24

**Authors:** Takeshi Machida, Akiko Ishibashi, Ai Kirino, Jun-ichi Sato, Shinji Kawasaki, Youichi Niimura, Ken-ichi Honjoh, Takahisa Miyamoto

**Affiliations:** 1 Department of Bioscience and Biotechnology, Faculty of Agriculture, Graduate School, Kyushu University, Fukuoka, Japan; 2 Department of Immunology, School of Medicine, Fukushima Medical University, Fukushima, Japan; 3 Department of Bioscience and Biotechnology, Graduate School of Bioresource and Bioenvironmental Sciences, Kyushu University, Fukuoka, Japan; 4 Department of Bioscience, Tokyo University of Agriculture, Setagaya-ku, Tokyo, Japan; Instituto de Biociencias - Universidade de São Paulo, Brazil

## Abstract

Chloroplast NADPH-dependent thioredoxin reductase (NTRC) catalyzes the reduction of 2-Cys peroxiredoxin (2-Cys Prx) and, thus, probably functions as an antioxidant system. The functions of the enzyme in oxidative and salt stresses have been reported previously. We have previously identified and characterized NTRC in *Chlorella vulgaris*. In the present study, we isolated a full-length cDNA clone encoding 2-Cys Prx from *C. vulgaris* and investigated the involvement of *Chlorella* NTRC/2-Cys Prx system in several environmental stress tolerances by using yeast as a eukaryotic model. Deduced *Chlorella* 2-Cys Prx was homologous to those of chloroplast 2-Cys Prxs from plants, and two conserved cysteine residues were found in the deduced sequence. Enzyme assay showed that recombinant mature *C. vulgaris* NTRC (mCvNTRC) transferred electrons from NADPH to recombinant mature *C. vulgaris* 2-Cys Prx (mCvPrx), and mCvPrx decomposed hydrogen peroxide, *tert*-butyl hydroperoxide, and peroxynitrite by cooperating with mCvNTRC. Based on the results, the mCvNTRC/mCvPrx antioxidant system was identified in *Chlorella*. The antioxidant system genes were expressed in yeast separately or coordinately. Stress tolerances of yeast against freezing, heat, and menadione-induced oxidative stresses were significantly improved by expression of *mCvNTRC*, and the elevated tolerances were more significant when both *mCvNTRC* and *mCvPrx* were co-expressed. Our results reveal a novel feature of NTRC: it functions as an antioxidant system with 2-Cys Prx in freezing and heat stress tolerances.

## Introduction

Chloroplast NADPH-dependent thioredoxin reductase (NTRC) is an NADPH-dependent thioredoxin reductase (NTR) isozyme specifically found in photosynthetic organisms [1], [2]. It consists of two individual domains: an NTR domain in its N-terminus and a dithiol-disulfide oxidoreductase, thioredoxin (Trx), domain in its C-terminus. Thus, unlike other isoforms of NTR, NTRC can directly reduce a thioredoxin peroxidase, peroxiredoxin (Prx). NTRA (mitochondrial) and NTRB (cytosolic), by contrast, require specific Trx proteins to reduce Prx [3], [4]. Trx and Prx have been reported to serve as antioxidant enzymes by cooperating with NTR [5] and ferredoxin-thioredoxin reductase [6], and to protect cells and their subcellular components against oxidative damage by eliminating reactive oxygen species (ROS) in cells [7]. Given its antioxidant activities, the NTRC system is considered important machinery in plant chloroplasts. In addition, the observed coordination of the domains when present in a single polypeptide produces high-efficiency electron transfer to Prx and protects cells against salt, drought, and methyl viologen-induced oxidative stresses [1]. In recent years, Pérez-Ruiz and Cejudo [8] have described the molecular basis of NTRC activity. They demonstrated that reduction of the Trx domain is catalyzed not only by the NTR domain in the same polypeptide, but also intermolecularly by the NTR domain in a second polypeptide. The catalytic mechanism is different from that observed in bacterial AhpF, in which the Trx and NTR domains are also conjugated. In bacterial AhpF, the NTR domain reduces only the Trx domain in its own molecule [9]. NTRC exhibits many features that differ from other NTR isozymes and, therefore, warrants further investigation.

Several researchers have previously investigated the involvement of the NTRC/2-Cys Prx system in several stress conditions, such as cold temperature, methyl viologen, and heat, by using *Arabidopsis*, and they have found that NTRC and 2-Cys Prx could enhance some environmental stress tolerances [4], [10], [11]. The NTRC/2-Cys Prx system may enhance many other environmental stress tolerances because such stresses seem to be accompanied by oxidative stress [12]. Our previous study [9], [13] suggested the involvement of mCvNTRC in the acquisition of freezing tolerance of the unicellular green alga, *Chlorella vulgaris*, by interacting with *Chlorella* 2-Cys Prx. To clarify their involvement and function in the acquisition of freezing tolerance of *Chlorella*, in the present study, we newly isolated a cDNA clone encoding 2-Cys Prx from *Chlorella* and examined peroxide reduction activity of *Chlorella* NTRC/2-Cys Prx antioxidant system by reconstitution experiment using both recombinant mCvNTRC and mCvPrx proteins. Furthermore, the corresponding genes were expressed in yeast to investigate the effect of the mCvNTRC/mCvPrx antioxidant system on several environmental stress tolerances.

## Materials and Methods

### Strains and conditions

*Escherichia coli* BL21(DE3)pLysS/pET-29b(+)/*mCvNTRC* was used for expression of mature CvNTRC fused with a His-tag in its C-terminal end (His-mCvNTRC) [13]. His-mCvNTRC was expressed by incubation of the *E. coli* cultures at 25°C for 6 h in the presence of 1 mM isopropyl-ß-D-thiogalactopyranoside (IPTG) as described previously [13]. *E. coli* JM109 strain was used to express a mature form of the *Chlorella 2-Cys Prx* gene (*mCvPrx*). *Saccharomyces cerevisiae* YPH500 (*Mata*, *ura3-52*, *lys2-801^amber^*, *ade2-101^ochre^*, *trp1-Δ63*, *his3-Δ200*, *leu2-Δ1*; Stratagene, La Jolla, CA, USA) was used for expression of *mCvNTRC* and *mCvPrx* genes.

### Cloning and sequence analysis of a full-length cDNA clone encoding CvPrx

Poly(A)^+^ RNA was isolated from cells of *Chlorella vulgaris* hardened at 3°C for 24 h as described previously [13]. First-strand cDNA was synthesized using oligo (dT) primer with 3′-adaptor sequence (CDS III 3′-primer) and 5′-adaptor oligonucleotide (SMART IV Oligonucleotide), which were supplied in the SMART cDNA Library Construction Kit (Clontech, Mountain View, CA, USA). Partial fragment for *CvPrx* gene was amplified by PCR with primers (5′- TTC TTC TAC CCC CTG GAC TTC AC -3′ and 5′- GGC AGA GAA GTA CTC CTT GG -3′). The primers were designed based on two conserved regions of amino acid sequences of 2-Cys Prx from photosynthetic eukaryotes (Fig. S1), and nucleotide sequences of the primers were determined according to nucleotide sequence of *2-Cys Prx* gene from *Chlamydomonas reinhardtii* (AJ304856). The amplified fragment was subcloned into a pT7-blue vector (Takara, Kyoto, Japan) and the plasmid construct was transformed into *E. coli* followed by sequencing. The full-length sequence of *CvPrx* was determined by the rapid amplification of cDNA ends (RACE) method using adaptor-specific primers and gene-specific primers based on the obtained cDNA fragment. The RACE fragments were directly sequenced. The resulting sequences of the partial fragment and the RACE fragments were assembled to determine the full-length cDNA sequence. Sequence alignment and phylogenic analysis were performed using the ClustalW program (http://www.genome.jp/tools/clustalw/) and the TreeView program [14].

### Expression and purification of recombinant mCvPrx protein

A DNA region (from 161 to 745 bp) corresponding to mCvPrx protein was introduced into a pTrc99A expression vector to form a His-tagged protein (His-mCvPrx), and the construct was transformed into *E. coli* JM109. For the efficient expression of His-mCvPrx in *E. coli*, five rare codons for *E. coli* were modified as described in the Results section. His-mCvPrx was expressed by incubation of the *E. coli* cultures at 37°C for 3 h in the presence of 1 mM IPTG.

The *E. coli* cells cultured as described above were collected by centrifugation at 8,000× *g* for 5 min, and resuspended in 50 mM sodium-phosphate buffer (Na-Pi, pH 7.0) containing 1 mM phenylmethylsulfofluoride. The cells were disrupted at 4°C by sonication using a Tomy Ultrasonic Disrupter UP-201 (Tomy Seiko, Tokyo, Japan) for 20 min at 48 W with 0.5-s pulses at 0.5-s intervals. Then, the samples were centrifuged at 20,000× *g* for 10 min at 4°C to recover soluble protein extract. His-mCvNTRC was purified using a HisTrap FF crude column (GE healthcare, Little Chalfont, UK) according to the manufacturer's instructions, and directly subjected to a PD-10 desalting column (GE healthcare) to remove imidazole in the eluate. His-mCvPrx was purified using a HisTrap FF crude column and the eluate was treated with 400 µM H_2_O_2_ for 1 h on ice to oxidize thiol (−SH) groups of the protein completely. The treated sample was then subjected to a PD-10 desalting column to remove imidazole and H_2_O_2_ in the eluate. Polyclonal anti-mCvPrx antibodies were raised in rabbits using purified His-mCvPrx as an antigen.

### Peroxide reduction assay

Assays were performed in double beam mode by using an UV-visible spectrophotometer DU800 (Beckman coulter, Brea, CA, USA). ROS-scavenging activities of a protein mixture of His-mCvNTRC and His-mCvPrx against hydrogen peroxide (H_2_O_2_), *tert*-butyl hydroperoxide (*t*-BOOH), peroxynitrite (ONOO^-^), and decomposed ONOO^−^ (PNdec) were determined by an enzymatic assay [15]. The enzymatic assay was performed by monitoring the decrease in absorbance at 340 nm due to NADPH oxidation. The assay mixture contained 1 mM EDTA, 100 µM peroxide substrates, 300 µM NADPH, 4.0 µM His-mCvPrx, and 4.0 µM His-mCvNTRC in 1 mL of 50 mM Na-Pi (pH 7.0). Measurement of A_340_ was started immediately after the addition of peroxide substrates. The initial rate of NADPH oxidation was calculated from the slope between 0 and 5 s after the addition of the peroxide substrate and corrected for the background oxidation of NADPH observed in the coupled assay without protein.

Peroxynitrite was purchased from Cayman Chemical (Ann Arbor, MI, USA), diluted with 0.3 mM NaOH, and stored at −80°C until use. The precise concentration of peroxynitrite was determined from the absorbance at 302 nm according to the manufacturer's instruction (Cayman chemical). PNdec was prepared by incubating peroxynitrite diluted in 50 mM NaPi (pH 7.0) for 5 min at room temperature. Decomposition of ONOO^−^ was confirmed by measuring A_302_ of the incubated solution.

### Plasmid construction and transformation of yeast

The DNA region encoding mCvNTRC was introduced downstream of a GAL1 promoter (galactose-inducible), and the region encoding mCvPrx was introduced downstream of a GAL10 promoter (galactose-inducible) in a pESC-TRP vector (Stratagene), subsequent to PCR amplification and restriction enzyme digestion of the fragments. The constructed plasmids were transformed into YPH500 by the lithium acetate method [16] to construct three yeast transformants, designated pNTRC (expressing *mCvNTRC*), pPrx (expressing *mCvPrx*), and pNTRC/Prx (expressing both *mCvNTRC* and *mCvPrx*). An empty vector was also transformed into YPH500 to construct a control strain, designated pESC.

### Expression of introduced genes in yeast and Western blot analysis

Yeast cells were cultured in SR medium (0.67% yeast nitrogen base, 2% raffinose, 0.2% complete supplement mixture) that lacked tryptophan (SR-Trp) and that contained 2% galactose, which induces expression of genes introduced downstream of GAL1 and GAL10 promoters. The cultured cells were collected by centrifugation, and the pelleted cells were resuspended in 50 mM Na-Pi (pH 7.0). The cell suspension was homogenized with an equal volume of 0.5 mm diameter glass beads for 5 min with a vortex mixer. It was then centrifuged at 20,000× *g* for 10 min at 4°C, and the supernatant was recovered as a protein extract.

The protein extracts of yeast transformants were subjected to SDS-PAGE on a 12% polyacrylamide gel [17], and the separated proteins were electroblotted onto a nitrocellulose membrane [18]. The expressed proteins on the membrane were detected with rabbit anti-mCvNTRC antibodies [13] and rabbit anti-mCvPrx antibodies according to the method of Machida *et al.* [13]. Goat anti-rabbit IgG peroxidase-conjugate (Sigma, St. Louis, MO, USA) was used as a secondary antibody.

### Stress tolerance test

Yeast cells were cultured in SR-Trp containing 2% galactose to exponential phase (OD_600_ = 0.4–0.6). The exponential culture was centrifuged, and the pelleted cells were washed twice with 0.9% NaCl and then were resuspended in 0.9% NaCl at the concentration of 2–3×10^6^ cells/mL (OD_600_ = 0.1). Freezing treatment was performed as follows. An aliquot of the suspension (100 µL) was transferred to a microcentrifuge tube, placed in a deep-freezer at −80°C for 2 min for ice formation, and then stored in a freezer at −20°C for 24 h. Heat stress treatment was performed as follows. An aliquot of the suspension (1 mL) was transferred to a test tube and incubated at 48°C in a water bath for 1 h with shaking. Oxidative stress treatment was performed as follows. Menadione (20 mM, dissolved in 100% EtOH) was added at a final concentration of 50 µM to 1 mL of the suspension in 100 mM Na-Pi (pH 7.0, OD_600_ = 0.1). Then, the mixture was transferred to a test tube and incubated at 30°C for 3 h with shaking. After treatment, cells were appropriately diluted with 0.9% NaCl (for freezing and heat stresses) or 100 mM Na-Pi (for oxidative stress) and plated onto YPD agar plates (1% yeast extract, 2% peptone, 2% dextrose, and 1.5% agar) followed by incubation at 30°C for 2 d. Viability (%) was calculated by dividing the counts of stressed cells on YPD agar plates by that of unstressed cells.

### Detection of intracellular ROS by fluorescence microscopy

To evaluate the effect of the mCvNTRC/mCvPrx antioxidant system in the transformed yeast, intracellular ROS were detected with dihydroethidium (Molecular Probes, Invitrogen, Karlsruhe, Germany), which specifically detects superoxide anion. Dihydroethidium was dissolved in 100% dimethylsulfoxide (DMSO) and added to the cell suspension in 100 mM Na-Pi (pH 7.0) already containing 50 µM menadione at a final concentration of 2 µM. The final concentration of DMSO in the suspension was never higher than 0.1%. The suspension containing dihydroethidium was incubated at 30°C for 3 h with shaking. The treated cells were harvested and mounted onto a slide glass. Fluorescence in yeast cells was visualized using a confocal laser-scanning microscope ECLIPSE E600 (Nikon, Tokyo, Japan) with excitation wavelength at 510–550 nm and emission wavelength >590 nm.

### Statistical analysis

Statistical analysis was performed using GraphPad Prism 5 software for Mac OS X (GraphPad Software, San Diego, CA, USA). Single groups were compared by unpaired two-tailed *t* test. One-way analysis of variance followed by Tukey's test was used for multiple comparisons. A *p*<0.05 was considered significantly different.

## Results

### Isolation of a full-length cDNA encoding 2-Cys Prx from *Chlorella*

**Figure 1:**
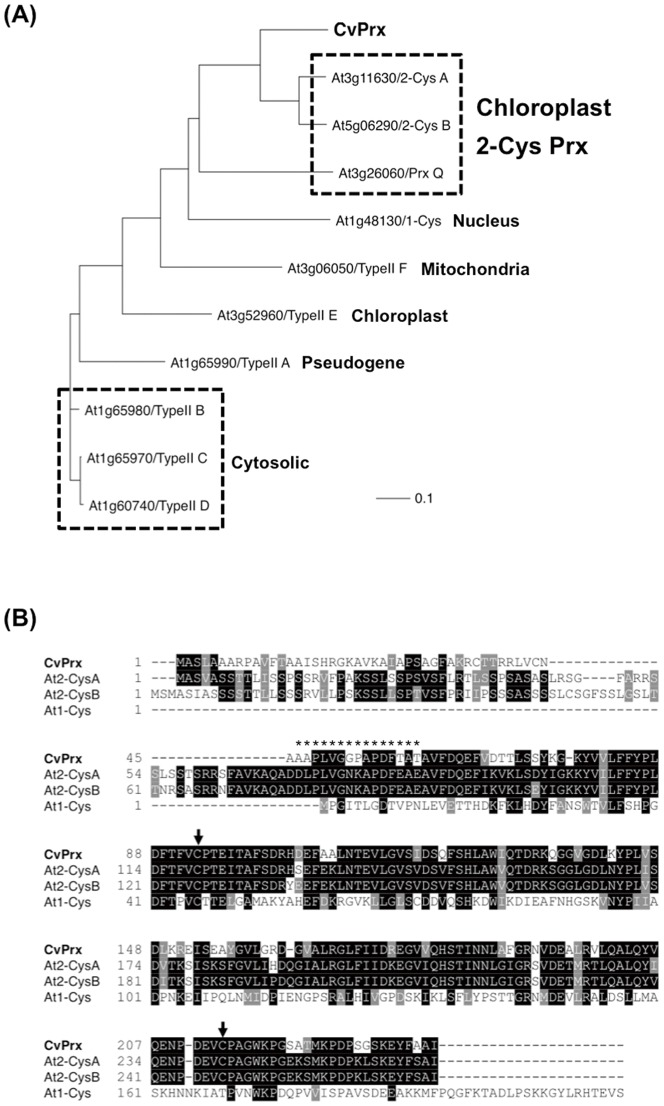
Sequence analyses of the full-length cDNA corresponding to *CvPrx*. (A) The phylogenetic tree was constructed with the full-length deduced amino acid sequences of *Prx* genes from *Arabidopsis* and *Chlorella* using the Clustal W program with bootstrapping, and was made visible with the TreeView program. Bar, 0.1 amino acid substitutions per site. (B) The amino acid sequence deduced from *CvPrx* was compared with the sequences deduced from *Arabidopsis Prx* cDNAs. The arrows indicate two conserved cysteine residues found in 2-Cys Prx. The asterisks indicate the N-terminal sequence of predicted *Chlorella* Prx described previously [13].

A partial cDNA fragment corresponding to *Chlorella* 2-Cys Prx was amplified using two primers as shown in Materials and methods. The amplified product was 425 bp in length, and the deduced amino acid sequence was homologous to those of plant *2-Cys Prx*s (data not shown). The full-length *CvPrx* cDNA determined by RACE was 868 bp in length and encoded 239 amino acids, which were deposited at DDBJ/EMBL/GenBank, under accession number AB682671. The deduced amino acid sequence of *CvPrx* showed homology to the deduced amino acid sequences of the chloroplast *2-Cys Prx* genes from *Arabidopsis*, which are characterized by the two conserved cysteine residues (Fig. 1). The N-terminal sequence of a predicted mCvPrx protein isolated by pull-down assay [13] was used to determine the DNA region encoding mCvPrx: from 161 to 745 bp corresponding to 194 amino acids. The upstream region, from 26 to 160 bp, is perhaps a region encoding a chloroplast transit peptide.

### Expression of recombinant mCvPrx protein in *E. coli*

**Figure 2:**
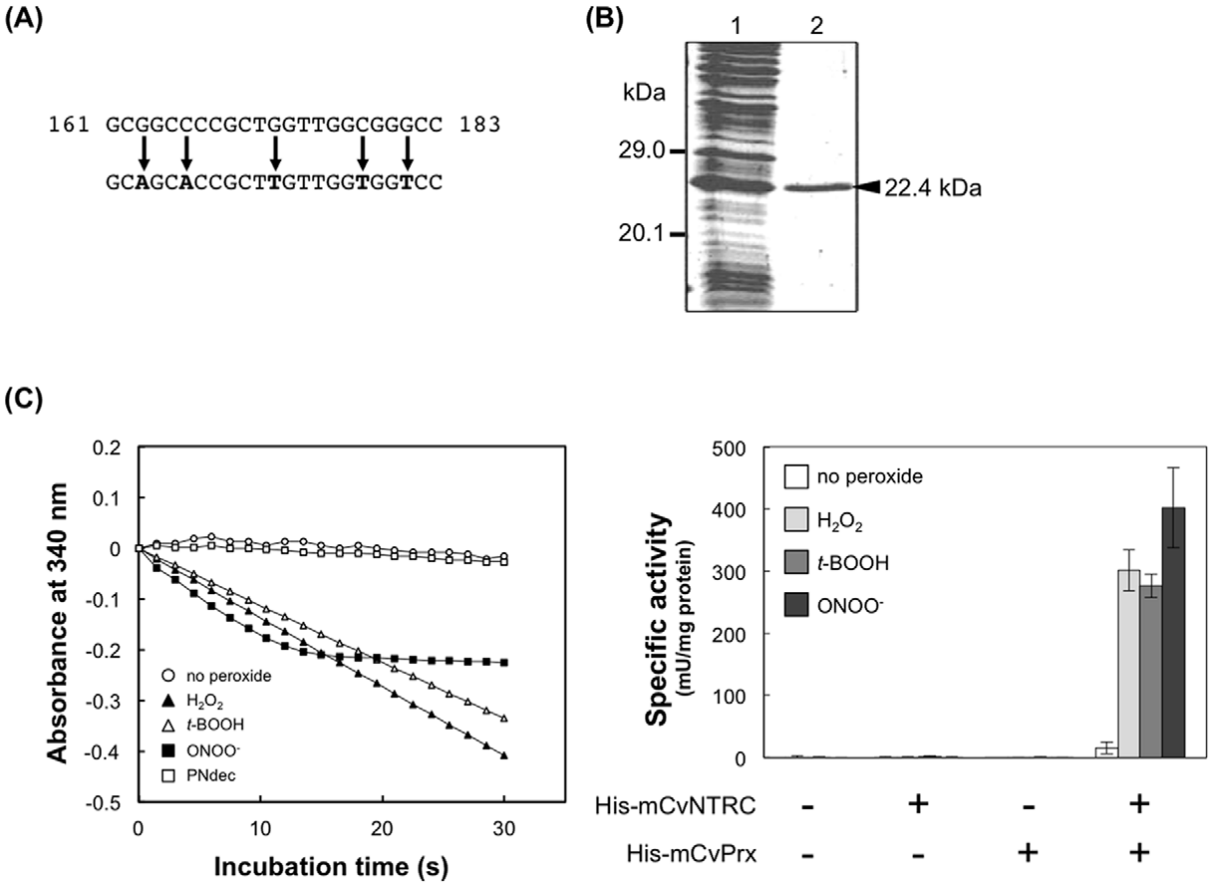
Peroxide reduction activity of His-mCvNTRC and His-mCvPrx proteins expressed in *E. coli*. (A) Five nucleotides in *mCvPrx* were modified for efficient expression of mCvPrx in *E. coli*. The original sequence is shown above, and the modified sequence is shown below. (B) His-mCvPrx was expressed in *E. coli* and purified by Ni^2+^-affinity chromatography. Lane 1, soluble protein extract of *E. coli*; lane 2, expressed protein purified by affinity chromatography. The arrowhead indicates a band for the His-mCvPrx. (C) *(left)* Representative kinetic data of peroxide reduction activities of mCvNTRC/mCvPrx against three peroxide substrates and decomposed peroxynitrite (PNdec) were shown. *(right)* Peroxide reduction activity of mCvNTRC/mCvPrx was shown as specific activity. Values are mean ± SD obtained from four independent experiments.

To determine the protein encoded by the *CvPrx*, a cDNA region encoding mCvPrx, which was modified as shown in Fig. 2A for the efficient expression in *E. coli*, was introduced into a pTrc99A vector and expressed in *E. coli* as a His-tagged protein. His-mCvPrx protein was purified and analyzed by SDS-PAGE (Fig. 2B). His-mCvPrx protein was 22.4 kDa in size, which was closely in accordance with predicted molecular size of deduced His-mCvPrx protein (22.5 kDa).

### Peroxide reduction activity of recombinant mCvNTRC and mCvPrx proteins

Peroxide reduction activities against three peroxide substrates were assayed with purified recombinant His-mCvNTRC and His-mCvPrx proteins. As shown in Fig. 2C, they showed no or only a slight NADPH oxidation when tested individually, whereas a mixture of both proteins showed a remarkably high rate of NADPH oxidation against both H_2_O_2_ and *t*-BOOH, suggesting that they did not function individually but reduced peroxides coordinately in the presence of NADPH. Furthermore, the mCvNTRC/mCvPrx antioxidant system could decompose ONOO^−^, while no NADPH oxidation was detected in the presence of PNdec (Fig. 2C), indicating it decomposed ONOO^−^ but not any product of ONOO^−^ decomposition. Rate of NADPH oxidation was much faster in the mixture with peroxide substrates than in the mixture without peroxides (Fig. 2C), indicating that the mCvNTRC/mCvPrx system could reduce peroxides directly. We also measured antioxidant activity of mCvNTRC/mCvPrx system in the presence of NADH as a cofactor, and no or slight NADH oxidation was detected (data not shown), suggesting that the mCvNTRC/mCvPrx system used NADPH as a specific coenzyme to reduce peroxides, similarly to the feature of other plant NTRC [1], [25].

### Expression of *mCvNTRC* and *mCvPrx* in yeast

**Figure 3:**
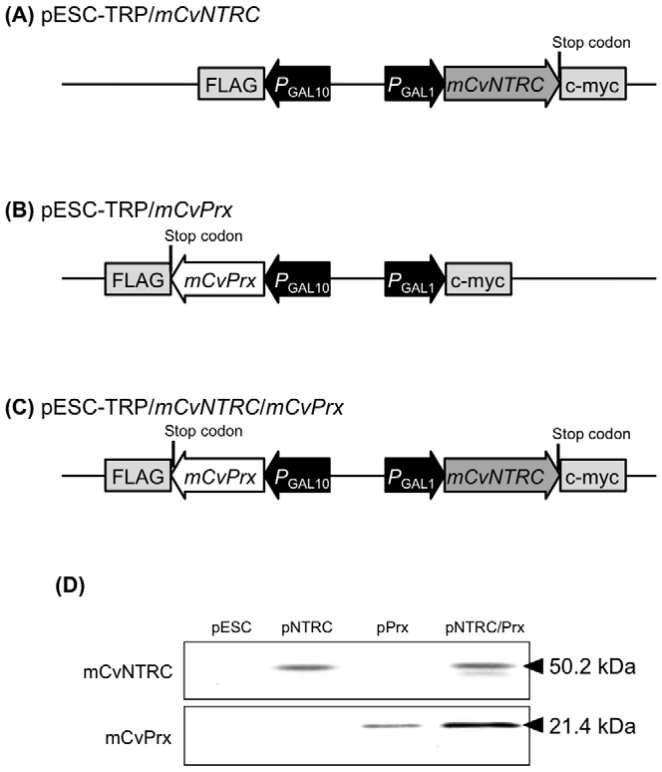
Expression of mCvNTRC and mCvPrx in yeast. Structure of plasmids for expression of (A) *mCvNTRC*, (B) *mCvPrx*, and (C) both genes in yeast. (D) Expression of mCvNTRC and mCvPrx proteins was checked by Western blot analyses. The arrowheads indicate bands for the proteins of interest in each blot.

To express *mCvNTRC* and *mCvPrx* in yeast, the corresponding genes were introduced into a pESC-TRP vector (Fig. 3A–C), and the constructs were transformed into yeast YPH500. A transformant carrying an empty pESC-TRP vector was also constructed as a control strain. Expression of the introduced genes were induced by incubation in galactose-containing medium and then confirmed by immunoblot analyses (Fig. 3D). Molecular masses of the expressed proteins were fairly identical to those predicted from the deduced amino acid sequences (mCvNTRC: 50.2 kDa, mCvPrx: 21.4 kDa).

### Stress tolerance of transformed yeast expressing *mCvNTRC* and *mCvPrx*

**Figure 4:**
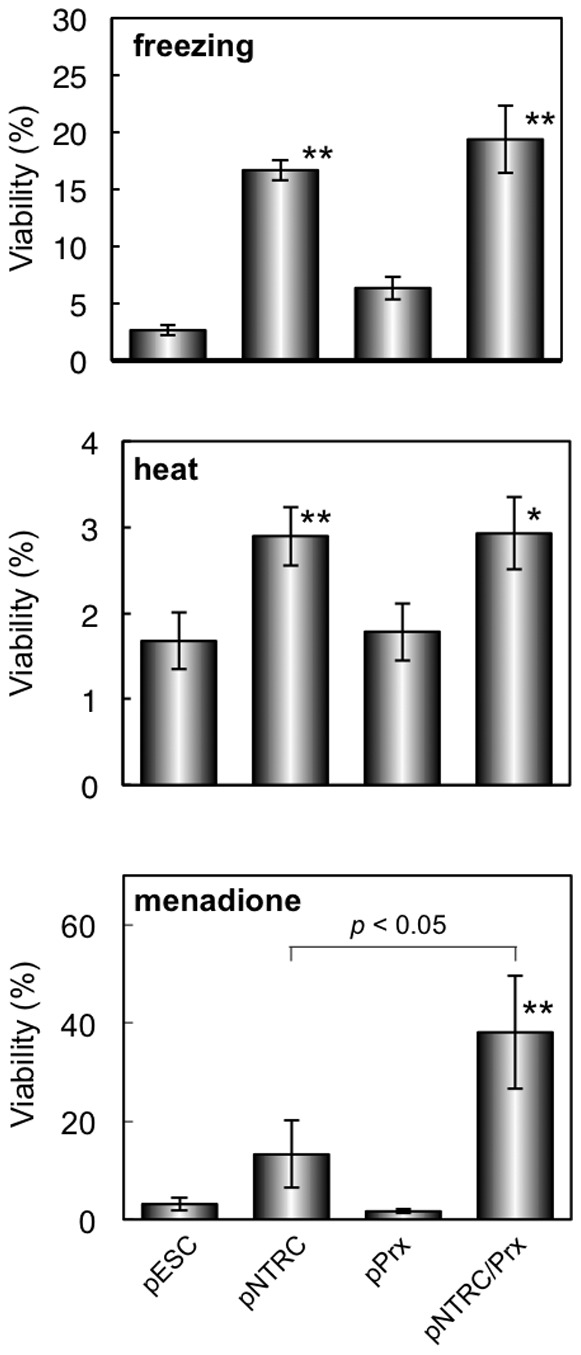
Stress tolerances of yeast against freezing (top), heat (middle), and menadione-induced oxidative (bottom) stresses. Values are mean ± SD obtained from three or four independent experiments. Significance against pESC is indicated as *p<0.01; **p<0.001).

To investigate the effect of the mCvNTRC/mCvPrx antioxidant system on environmental stress tolerances, the yeast transformants were subjected to freezing (−20°C, 24 h), heat (48°C, 1 h), and manadione-induced oxidative (50 µM, 30°C, 3 h) stress conditions. As shown in Fig. 4, freezing and heat stress tolerances were significantly improved in pNTRC and pNTRC/Prx strains, while those in pPrx strain were not improved. Menadione-induced oxidative stress tolerance of yeast was significantly improved only in pNTRC/Prx strain. Furthermore, there was a significant difference in oxidative stress tolerance between pNTRC and pNTRC/Prx, although their viabilities were not different significantly against freezing and heat stresses.

### Superoxide levels in yeast cells generated during menadione treatment

**Figure 5:**
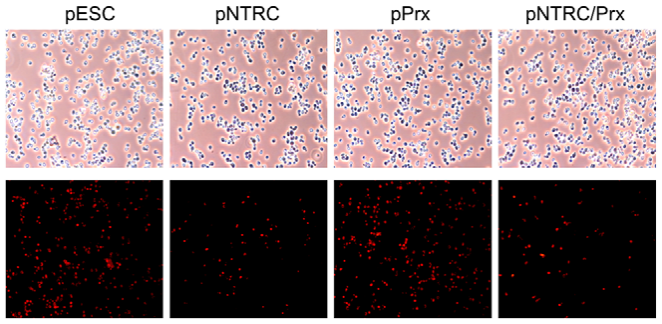
Superoxide generated in yeast cells during menadione treatment. Upper photos were taken with phase contrast microscopy, and lower photos were taken with fluorescence microscopy. Intensity of red fluorescence, which is derived from dihydroethidium, indicates superoxide levels in cells.

Superoxide in yeast cells during menadione treatment was detected by fluorescent microscopy using dihydroethidium as a detection reagent for superoxide anion radicals. As shown in Fig. 5, fluorescence intensity was partly less in pNTRC and pNTRC/Prx strains against control, while the intensity in pPrx was almost the same as that in pESC, suggesting less superoxide accumulation in pNTRC and pNTRC/Prx strains.

## Discussion

NTRC is involved in many cellular reactions, such as a cellular protection against stresses (oxidative, drought, and salt), starch synthesis, and photoperiodic development [1], [19]–[21]. In addition to such functions, we found a possible novel function of NTRC in the acquisition of freezing tolerance of *C. vulgaris* C-27, a frost-hardy strain [13], [22]. Although NTRC has been reported to function as an antioxidant with 2-Cys Prx in plants, the system has not yet been identified in *Chlorella*. Neither 2-Cys Prx nor any type of *Chlorella* Prx has been isolated and investigated to our knowledge. Moreover, a cell-free extract of *Chlorella* did not exhibit reduction activity against *Synechocystis* Prx (*sll0755*) in the presence of NADPH [23]. Prx was reduced in the presence of NADH [23], however, which implied that *Chlorella* did not have an NADPH-dependent reduction enzyme for Prx. Contrary to the report, information published in the DOE Joint Genome Institute database (http://genome.jgi-psf.org/) suggested that NTRC and 2-Cys Prx are probably encoded in the *Chlorella* genomic DNA. To characterize the antioxidant system in *Chlorella*, we first isolated a full-length cDNA sequence of *CvPrx*, and determined *mCvPrx* region based on the N-terminal sequence of mCvPrx protein identified previously [13].

Plants have many isotypes of Prx proteins, as there are at least 10 isotypes of Prx in *Arabidopsis* [24]. Among them, plant NTRC can catalyze the transfer of electrons to a few specific Prx proteins [25]. In *Chlorella* cells, mCvPrx was identified as a partner protein of mCvNTRC using *in vitro* pull-down assay [13], but the cooperating antioxidant activity of the system was not demonstrated. To assess the antioxidant capacity of the mCvNTRC/mCvPrx system and the direct interaction between them, His-mCvPrx protein was prepared using an *E. coli* expression system. Enzyme assay indicated that His-mCvPrx showed cooperating antioxidant activity specifically observed in the presence of His-mCvNTRC (Fig. 2C). Together with previous reports, our results suggest that mCvNTRC functions as a specific electron donor to mCvPrx, although other Prx types have not been identified in *Chlorella* and investigated. Furthermore, our results for the first time demonstrate the ONOO^−^ scavenging activity of the plant NTRC/2-Cys Prx antioxidant system (Fig. 2C). Although Sakamoto *et al.* [26] showed using preliminary-reduced Prx that *Arabidopsis* 2-Cys Prx functioned in ONOO^−^ reduction, the experiment was not sufficient to explain the ONOO^−^ scavenging activity of the NTRC/2-Cys Prx system, since there are cases when the Trx domain of NTRC does not show activity mediated by intrinsic NTR domain even in the presence of NADPH [1], [13]. Thus, our results are the first to indicate the wide antioxidative spectrum of the mCvNTRC/mCvPrx antioxidant system, which may contribute to the broad range of environmental stress tolerances as well as to freezing tolerance of *Chlorella*.

In physiological studies of plant NTRC, Serrato *et al.* [1] and Perez-Ruiz *et al.* [4] have shown that NTRC is essential for plant development under abiotic stress conditions using the *Arabidopsis ntrc* mutant. Thus, it is strongly suggested that overexpression of NTRC can be utilized to confer several environmental stress tolerances to stress-sensitive plants. Previous studies on the involvement of NTRC and 2-Cys Prx in stress tolerance [1], [4], [27] suggest that the combined effect of NTRC and 2-Cys Prx on stress tolerance is stronger than the effect of NTRC alone, since the NTRC/2-Cys Prx antioxidant system functions in detoxification of ROS, which is generated under many stress conditions. In the present study, the effects of mCvNTRC, mCvPrx, and the mCvNTRC/mCvPrx antioxidant system on freezing, heat, and oxidative stress tolerances were investigated using yeast as a eukaryotic model. Yeast transformant pNTRC/Prx (expressing both genes) showed significantly high stress tolerances against freezing, heat, and menadione-induced oxidative stress conditions (Fig. 4). Furthermore, the intracellular superoxide level of the pNTRC/Prx strain was greatly reduced compared with superoxide level of the control pESC strain (Fig. 5) when examined under menadione-induced oxidative stress. There is some evidence that 2-Cys Prx functions in detoxification of H_2_O_2_ and alkyl hydroperoxide [28], but to our knowledge, there is no evidence that 2-Cys Prx reduces superoxide. Contradictorily, we could not detect H_2_O_2_ reduction in the pNTRC/Prx strain during menadione treatment when examined with carboxy-2′,7′-dichlorofluorescein diacetate (CDCFDA), a fluorescent dye for specific detection of H_2_O_2_ and alkyl hydroperoxide (data not shown). When organisms are exposed to superoxide anion, many oxidation reactions occur inside the cells and the intracellular superoxide is converted to peroxynitrite through the oxidation reactions [29]. Given the phenomenon and our results, mCvNTRC/mCvPrx antioxidant system might serve to decompose peroxynitrite, and result in reduction of intracellular superoxide level and improvement of menadione-induced oxidative stress tolerance of yeast pNTRC/Prx strain.

In addition, freezing and heat stresses were also alleviated in yeast expressing *mCvNTRC* against control (Fig. 4). These results suggest that mCvNTRC serves as an activator of the yeast intrinsic thioredoxin-dependent defense system (e.g., Tsa1, Tsa2, Ahp1) and alleviates freezing and heat stresses. Expression levels and activities of intrinsic yeast proteins that may interact with mCvNTRC should be investigated in further studies.

In the present study, we identified the NTRC/2-Cys Prx system in a freezing-tolerant *Chlorella* strain. We showed the capacity of the NTRC/Prx system to alleviate freezing stress, a finding that suggests the NTRC/Prx system may be of great importance in various plant stress tolerances. Further studies, including those to identify enzymes activated by the system and to further investigate relationships between the association state of 2-Cys Prx and environmental stress tolerance, may lead to elucidation of mechanisms for plant stress tolerance and effective strategies for conferring stress tolerance to plants.

## Supporting Information

Figure S1 **Alignment of amino acid sequences of 2-Cys Prx proteins from photosynthetic eukaryotes.** Accession numbers or loci for the sequences, shown in parentheses, are as follows: *O. locimarinus*-1: *Ostreococcus lucimarinus* CCE 9901 predicted protein (ABO97759), *O. locimarinus*-2: *O. lucimarinus* CCE 9901 predicted protein (ABP01316), *V. carteri* Prx1f: *Volvox carteri* f. *nagariensis* female Prx1 (ADI46867), *V. carteri* Prx1m: *V. carteri* f. *nagariensis* male Prx1 (ADI46952), *C. reinhardtii*: *Chlamydomonas reinhardtii* 2-Cys Prx (CAC19676), *A. thaliana* 2-Cys A: *Arabidopsis thaliana* 2-Cys Prx A (At3g11630), *A. thaliana* 2-Cys A: *Arabidopsis thaliana* 2-Cys Prx B (At5g06290). The asterisks indicate two conserved regions used for primer design for amplification of partial *CvPrx* cDNA fragment.
(TIF)
