# Supplementary figures and images for: Chloroplast NADPH-Dependent Thioredoxin Reductase from Chlorella vulgaris Alleviates Environmental Stresses in Yeast Together with 2-Cys Peroxiredoxin
Source: PLoS One. 2012 Sep 24;7(9):e45988. doi: 10.1371/journal.pone.0045988 (PMC3454380; doi:10.1371/journal.pone.0045988)

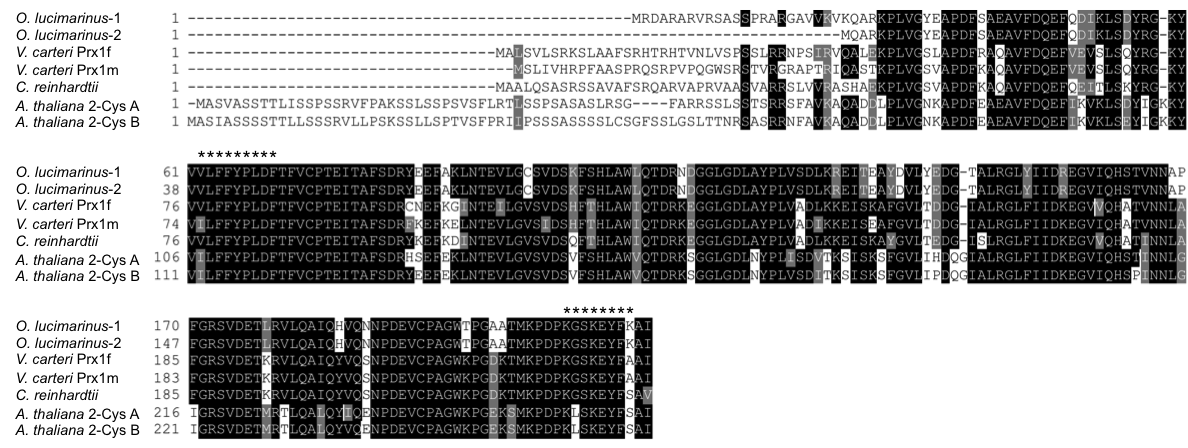

Supplement: Figure S1 — Alignment of amino acid sequences of 2-Cys Prx proteins from photosynthetic eukaryotes. Accession numbers or loci for the sequences, shown in parentheses, are as follows: O. locimarinus-1: Ostreococcus lucimarinus CCE 9901 predicted protein (ABO97759), O. locimarinus-2: O. lucimarinus CCE 9901 predicted protein (ABP01316), V. carteri Prx1f: Volvox carteri f. nagariensis female Prx1 (ADI46867), V. carteri Prx1m: V. carteri f. nagariensis male Prx1 (ADI46952), C. reinhardtii: Chlamydomonas reinhardtii 2-Cys Prx (CAC19676), A. thaliana 2-Cys A: Arabidopsis thaliana 2-Cys Prx A (At3g11630), A. thaliana 2-Cys A: Arabidopsis thaliana 2-Cys Prx B (At5g06290). The asterisks indicate two conserved regions used for primer design for amplification of partial CvPrx cDNA fragment. (TIF) [file pone.0045988.s001.tif]
